# Children’s perspective-taking and decision-making on forests and land use

**DOI:** 10.1186/s43031-025-00130-2

**Published:** 2025-05-27

**Authors:** Mijung Kim, Nimrah Ahmed, Kadriye Akdemir, Suzanna Wong, Okan Bulut

**Affiliations:** Faculty of Education, University of Alberta, 11210-87 Ave. Edmonton, Edmonton, AB T6G 2G5 Canada

**Keywords:** Socioscientific, Environmental, Decision making, Perspective taking, Climate change education

## Abstract

Students’ reasoning and decision making on complex socioscientific issues are critical for developing scientific literacy for 21st century citizenship. By incorporating a scenario-based approach, this study aims to understand the complexity of students’ decision making on environmental issues: forests and land use. To help students grasp the context of these issues, we developed scenarios reflecting their experiences and understanding of forests within local communities. Through scenario-based surveys, students in Grade 5–6 science classrooms were encouraged to explore diverse stakeholders’ perspectives and articulate their decisions regarding the scenarios. Additionally, students in focus groups participated in semi-structured discussions and interviews. The data collected from the surveys and students’ dialogues were thematically analyzed. The study found that students prioritized environmental concerns, demonstrated skepticism toward politicians’ perspectives, and emphasized righteousness in their decision making. These findings suggest that a holistic approach is essential to engage students’ diverse perspectives in socioscientific and environmental problem solving. However, this also highlights the ongoing challenge of disciplinary boundaries within school curricula and pedagogical practices in science classrooms.

## Introduction

### Students’ reasoning and problem solving skills on socioscientific issues

In science classrooms, scientific reasoning and problem solving skills emphasize students’ ability to understand and evaluate the coordination of claims and evidence in order to justify solutions (Kuhn, 2002; Kuhn et al., 2008). Students make and justify their claims with evidence and need to understand how certain evidence can support, verify, and refute their claims in problem solving processes. In well-defined problem contexts such as scientific investigations in classrooms, students are encouraged to collect and examine valid data and to analyze them for explanations and conclusions (Jeong et al., 2007). Well-defined problems often include empirical data, scientific theories, and laws to provide a degree of certainty, which helps students as problem solvers to understand and justify the relationship between evidence and claims (Wirth & Klieme, 2003). However, when problems related to everyday experiences that go beyond the epistemic boundaries of scientific knowledge stated in the curriculum, the problem solving process gets complex and becomes intertwined with other knowledge systems, such as personal, social, and cultural beliefs, values, and knowledge. Everyday socioscientific problems and environmental problems are ill-defined, complex, and controversial in nature. Most claims and decisions on everyday-related complex problems cannot be characterized or justified by the rules of the formal logic of reasoning to determine the truth or falseness of a proposition as multiple knowledge, perspectives, uncertainties of opportunities, risks, and values are inherent and complexly intertwined in inquiring, reasoning and decision making (Christenson et al., 2014; Dawson & Carson, 2020; Develaki, 2008; Kim et al., 2014; Zeidler & Sadler, 2011). The nature of knowledge and evidence gets challenged by diverse knowledge systems in those problems and challenges problem solvers with uncertainty and questions about what counts as knowledge and evidence in decision making and problem solving (Xun & Land, 2004). For instance, in health sciences, decisions on health issues are always challenging, with doubting evidence, distrust, and complexity of values. People’s decisions and actions do not prioritize empirical evidence over other knowledge and values, questioning what constitutes evidence and whose evidence is taken in public health issues (Rose et al., 2006).

As citizens face increasingly complex and challenging science-related issues in their everyday lives, questions have arisen about the meaning of scientific literacy, the characteristics of scientifically literate individuals, and how such literacy can be achieved through school science education (Roberts, 2011). Vision I of scientific literacy emphasized the scientific knowledge and skills necessary for success in scientific fields. However, this heavy focus on content was criticized for disconnecting science from students’ everyday experiences and real-world contexts. In response, Vision II of scientific literacy shifted the emphasis toward the relevance of science in everyday and societal situations encountered by students, parents, and citizens. This approach highlights the importance of applying scientific reasoning and skills to personal and social issues, strengthening the connection between science education and real-life problem solving (Roberts, 2011). The distinction between “pipeline science” (preparing students for scientific careers) and “science for all” (promoting broader scientific literacy) became a central focus of this debate (Sjöström & Eilks, 2018). With challenges of complex relationships among science, technology, and societal changes in one’s decision making, logic-based scientific reasoning becomes challenged when scientific knowledge gets intertwined with other knowledge systems. Vision III of Scientific Literacy (Sjöström, 2018; Valladares, 2021) extends students’ competence of scientific understanding and skills in social, cultural, political, and environmental contexts (Roberts, 2011; Siarova et al., 2019; Yore, 2012). Vision III emphasizes science for transformation of both students/citizens and society towards sustainability. Critical and reflexive thinking and reasoning for problem solving are critical for the transformation of individual and social relations (Sjöström & Eilks, 2018).

Pedagogical approaches such as STSE (Science, Technology, Society and the Environment) or SSI (SocioScientific Issues) framework have attempted to extend Vision I and II of scientific literacy to Vision III by engaging controversial scientific issues in society that involve perspectives, values, and ethics for critical, responsible, and reflexive reasoning (Bencze et al., 2020; Pedretti et al., 2008; Kinslow et al., 2019; Zeidler & Sadler, 2011). The STSE movement began with a practical focus on students’ experiences and their understanding of the relationships among science, technology, and society, aiming to address the demands of scientific literacy. Over the decades, the STSE movement has expanded to include sociocultural, political, and ethical dimensions, incorporating value-centered approaches and emphasizing socio-ecojustice in science education (Pedretti & Nazir, 2011). Similarly, the SSI approach highlights moral and ethical concerns by addressing complex and controversial everyday issues, aiming to transform pedagogy in science teaching (Sadler, 2004; Zeidler et al., 2011). While engaging with reasoning and problem solving on STSE or SSI topics, students’ beliefs, knowledge, and values are often challenged by the multifaceted nature and complexity of these issues. However, this approach helps develop students’ skills in evaluating and making decisions about competing scientific claims in real-world contexts (Zeidler et al., 2011). Transformation in science science teaching through the STSE and SSI approaches aligns with Vision III of scientific literacy, although the movement has faced its share of challenges. Human subjectivity, such as beliefs, values, and ethics, is still resisted in many science classrooms under the positivistic and authoritarian dogma of science (Bazzul, 2016; Donnelly, 2004; Levispn, 2004; Reiss, 1999). Accordingly, the STSE or SSI approach is often tokenistic as an add-on and not seen as science but as social studies (Hughes, 2000; Kim et al., 2021; Pedretti, 2003; Pedretti et al., 2013). This tendency can be more evident under the pressure of standardized curriculum and assessment. The absence of human values in science classrooms raises major concerns about the calls for scientific literacy vision III for 21st-century citizenship. In contrast, students continue to encounter and experience the complexity, confusion, uncertainty, and dilemmas of decision-making on STSE/SSI problems in their everyday lives.

### Student learning on socioscientific and environmental issues

Researchers on socioscientific reasoning have emphasized that scientific knowledge does not significantly affect students’ decision making on complex everyday socioscientific issues, but their intuitions, values, and emotions do impact one’s understanding and decision making (Sadler et al., 2004; Sadler & Donnelly, 2006). That is because scientific evidence is not always given priority and scientific knowledge is often compartmentalized in everyday socioscientific problem solving (Sadler & Zeidler, 2005; Sund, 2015). Thus, it is critical to bring students’ epistemic framework and worldviews in reasoning and problem solving tasks in classrooms (Albe, 2008; Christenson & Walan, 2022; Hodson, 2013; Zeidler et al., 2005). During the tasks, students are encouraged to reflect and examine diverse knowledge, information, and experiences intertwined in the issues and ambiguous boundaries among them to understand the complexity of problem solving (Garrecht et al., 2020; Newton & Zeidler, 2020). Environmental issues being situated in socioscientific, economic, cultural and personal contexts require thoughtful reflections on available information, time and place-based problems and affective domains in one’s decision making. To develop effective environmental decision making, the complexity of multifaceted knowledge, perspectives, and other influential variables, such as value structures, experiences, and emotions, need to be addressed in classroom situations (Arvai et al., 2004; Sund, 2015). Based on these challenges and the complexity of decision making, this study aims to move beyond the deficit model, a linear approach to students’ reasoning and decision making on environmental issues.

A socioscientific reasoning (SSR) model suggests students’ thinking and decision making with the complexity of issues, diverse perspectives, and skeptical thinking in problem solving inquiry (Kinslow et al., 2019; Sadler et al., 2011; Zeidler et al., 2019). When students are asked to make decisions on socioscientific issues, they are encouraged to explore the complex and open-ended nature of the issues and examine multiple perspectives embedded in the issues with critical questions and inquiry. During students’ SSR, perspective taking is often related to affective domains that influence their decision making greatly (Arvai et al., 2004). Researchers explain perspective taking is a cognitive capacity to take the perspectives of others (Li et al., 2023) by recognizing and comprehending various thoughts, emotions, and intentions, often shaped by the surrounding context of the issues and also take their own perspectives for decision making (Vogel & Hunecke, 2024; Heasman & Gillespie, 2023). It encourages students to grasp the complexity of diverse knowledge systems, recognize conflicts between differing perspectives of others and understand the importance of evaluating and negotiating these differences (Berglund & Gericke, 2015). As students are taking others’ perspectives, views, and situations, it also engages students’ emotive reasoning, such as empathy or an appreciation of different viewpoints and moral reasoning to help others. When students take the perspectives of others who are suffering in problem situations, they feel empathetic and motivated to understand the problems and find ways to help solve problems. So it also engages emotive and moral reasoning, and thus, evokes altruistic motivation (Batson et al., 1997; Li et al., 2023). Together with critical, evidence-based reasoning, this is a critical component of scientific literacy for transformative citizenship (Kahn & Zeidler, 2019; Newton & Zeidler, 2020).

A scenario-based approach that uses stories or real-life scenarios has been implemented to help students learn complex content and contexts of scientific and environmental questions in lifeworlds (Albe, 2008; Berglund & Gericke, 2015; Sadler et al., 2011). This approach is particularly useful in educational settings where everyday informal reasoning with complex issues in communities is limited in classroom learning. When environmental issues manifest and impact one’s being and living in communities, a scenario-based approach helps students learn and understand the complexity of issues and decision making within the communities. Students engage in the scenarios by exploring and understanding the tension and difficulties of decision making on certain issues in a safe environment, while still being in a real-life situation (Jones, 2014). This approach helps develop a normative teaching tradition of science and environmental education into a pluralistic and more democratic approach to teaching and learning (Sund, 2015). However, while the benefits of scenario-based learning are well-documented, little research has investigated how students navigate and make decisions on scientific issues by following their thought processes. Further research is needed to understand how students reason and make decisions within real-life scenarios, thereby illuminating the steps they take in forming judgments on complex scientific matters.

In this study, we used a scenario-based approach to engage students in decision-making processes related to local environmental issues that involve diverse knowledge systems, perspectives, values, and social contexts. We aimed to explore how students understand, evaluate, and discuss these factors when making decisions. Given the limited understanding of the complexity of elementary students’ socioscientific and environmental reasoning and decision making, this study examines how they assess diverse knowledge, perspectives, and values, and how these assessments influence their decision making on environmental issues. Our research was guided by the following questions.

1. How do students evaluate stakeholders’ perspectives in the scenario?
2. How do students discuss and prioritize diverse perspectives in their decision making process?

By exploring the research questions, we aimed to understand the complexity of students’ perspective taking and decision making on socioscientific and environmental issues.

## Research process

### Local context

Providing students with opportunities to delve into multiple knowledge and value systems (scientific, sociocultural, emotional, and moral), we were interested in hearing students’ active voices in knowledge evaluation and negotiation in socioscientific reasoning practice, particularly perspective taking during their decision making. By incorporating a scenario-based approach, we attempted to encourage students to explore decision making processes on scientific and environmental issues in their local community. We developed a scenario incorporating multifaceted knowledge, perspectives, conflicts, and tensions related to forests and land use, often discussed alongside climate change issues. The scenario’s content and context were designed to engage students by connecting with their interests, familiarity, everyday experiences, and curriculum for the following reasons.

Firstly, the school is located in the province of Alberta where there are a lot of connections to the Rocky Mountains and forests. With approximately 60% of the province covered by forests (Alberta Wilderness Association, n.d.), discussions about land development are frequently related to forested areas. Many towns near the mountains are built around forests, making forests and land use deeply intertwined with community culture, decision making, and development. Students and their families visit the mountain sites for various activities. Recently, there have been increasing wildfire cases, which raised many concerns in the community. People nearby had to evacuate their homes for months, and many lost their homes because of wildfires. Vast areas of natural habitats were destroyed. Smoke traveled further into the city, creating air pollution and health concerns, impacting people’s everyday lives (Derworiz, 2019). The record-breaking number of wildfires in the province has sparked widespread public discussion, particularly in the context of climate change (Canadian Broadcasting Company News, 2024). The conservation of trees and forests is frequently addressed alongside issues of deforestation, land use, wildlife preservation, and sustainability.

Secondly, a survey poll conducted by a youth group in the province showed that the majority of youth (74%, grades 6–12) were worried about their future due to climate change (Alberta Youth Leaders for Environmental Education, 2021), while adults in the province showed the lowest level of concerns about human impact on climate change across Canada (Field et al., 2019; Gibson, 2018). There is a complex understanding of climate change in families and communities. Thirdly, climate change is part of the science curriculum. It was not explicitly stated as learning outcomes in the outgoing curriculum but is explicitly emphasized in the incoming curriculum (Alberta Education, 2021). In the outgoing curriculum, teachers often address climate change in the unit of Trees and Forests, which was the case in this research. Yet, teachers often experience challenges in teaching climate change in the science curriculum because of the demarcation between science and non-science content of climate change. As teaching climate change requires a holistic approach of diverse knowledge integration beyond the science of ecology (Reid, 2019), we implemented socioscientific reasoning and decision making processes to address climate change issues. The scenarios helped encourage students to reflect on their perspectives, knowledge, and local contexts while considering how to make informed decisions.

### Scenario development

With these backgrounds, a scenario (see Appendix A) was developed based on a true story that involved various stakeholders’ perspectives and debates surrounding land development near the mountains years ago. The scenario included the following ideas: a company suggests a plan to develop a multipurpose, amusement park (the Park hereafter) in a hypothetical town named Alfa, near the mountains. The plan indicated that trees would be cut in Alfa, and people nearby would move out to other communities. There are stakeholders (politicians, environmentalists, ecologists and biologists, sociologists, and parents) with different views to support or against the plan.

- Politicians believe that this business plan will turn Alfa into a world-renowned town. The company will pay a lot of taxes and make donations to develop education, social welfare, health care systems, etc.
- Environmentalists argue that climate change is becoming a serious problem, and deforestation has made climate change worse for many years. There has already been a lot of loss in forests from wildfires, logging, mining, etc., around the Rockies.
- Ecologists and biologists suggest reservation of the land in Alfa because it has a lot of value in biodiversity.
- Sociologists suggest that the displacement of local people in that region will be problematic. These people may lose their homes, communities, and cultures.
- Parents living in Alfa support the idea of having the resort and sports facilities in their town as it gives them a place to entertain their children. They believe this will provide children with many opportunities to explore winter and summer sports.

Students evaluated the stakeholders’ views using a 5-point Likert scale (ranging from “not important at all” to “very important”) and made their own decisions by voting on the plan with options of “yes,” “no,” or “not sure.” In the scenario, politicians emphasized tourism and economic benefits to justify their support for the development of the park. Ecologists and biologists highlighted the need to preserve the land due to its biodiversity. Environmentalists opposed the plan, stressing the impact of deforestation on climate change. Sociologists focused on how the park’s development would affect people’s lives and cultures in local communities around Alfa. Finally, parents supported the proposal, viewing it as an opportunity to provide entertainment and sports skills for children. This scenario was designed into a Google survey form. After students took the survey, they were engaged in three focus group sessions over a period of three weeks.

Based on the focus group students’ ideas and suggestions and classroom observations, we modified the initial scenario and developed a second scenario: the company considered students’ concerns and suggestions and made another proposal for the Park development. In the focus group discussions on the first scenario, students raised questions and critiqued the stakeholders’ perspectives and their credibility in making decisions about natural resources. Having prioritized the environment, students had already rejected the plan and started suggesting revisions to make the proposal more acceptable. To redirect their attention back to the diversity of perspectives and decision making process, we needed to revise and develop a second scenario. Therefore, we removed the titles of the five groups of stakeholders (politicians, environmentalists, ecologists and biologists, sociologists, and parents) in order to encourage students to analyze the development plan without any predefined ideas. The second survey suggested that only trees infected by mountain pine beetle attacks would be cut, there would be public transportation built for better access, indigenous peoples would benefit from this project and the construction company would pay taxes and invest in Alfa’s development. We followed this with individual interviews to understand each student’s decision making process and the perspectives they undertook to approach the revised survey (see Appendix B).

### Data collection in a case study

A case study was employed in this study. Putting a boundary of a specific case, a case study approach provides opportunities to explore the case in depth (Merriam & Tisdell, 2015). Due to the boundaries of a case, the case study approach is situational and embedded within a specific context (Baxter & Jack, 2008; Yin, 1994). This study examines the complexity of students’ perspectives and decision making processes regarding local environmental issues in science classrooms at a public school in Alberta. To understand the case, we collected diverse data sets such as classroom conversations and field notes, classroom videos, surveys, and semi-structured interviews and closely analyzed and interpreted students’ shared stories, experiences, and perspectives in relation to the local contexts. In this study, twenty-three Grade 5–6 students in a combined class were invited. The school, teacher, and participants were chosen through convenience sampling (Merriam & Tisdell, 2015). The school was located near the researchers’ university, and the teachers expressed interest in collaborating on the research. An invitation letter was sent to the school teachers, and one teacher agreed to participate in the study. Later, students in her class were invited to the study.

Throughout the study, students were engaged in learning the unit of trees and forests in science classrooms for 6 weeks. Students learned about various concepts of trees, forests, and ecosystems in general and local contexts and the values of trees and forests and human interactions with them. They also discussed sociocultural values and indigenous perspectives on nature in the classroom. Researchers observed science classes, taking observation notes. Classes were also video-recorded to cross-check the students’ learning and reasoning processes. We also collected data from Google surveys on scenarios, focus group discussions, and semi-structured individual interviews. There were two scenario-based surveys. At the beginning of the research, students completed the first survey to rate the importance of each perspective on a Likert scale and to explain their ratings. At the end of the survey, they indicated their final decision. The students completed the surveys in class, with the teacher assisting them in understanding the scenarios by clarifying terms and explaining the survey structure whenever they had questions. Based on the survey responses (students’ decisions and reasons for their decisions), we formed two focus groups of 5 students (2–3 students who supported the plan and 2–3 students who rejected the plan) for group discussion. To form focus groups, we shared students’ responses with the teacher and asked who could actively explain their thinking during group discussions. In the focus group discussions, students shared their understanding of the issues and stakeholders’ perspectives in the scenario and what needed to be thought further for better decision making. Getting toward the end of the unit, students took the second survey to explain their decisions. The focus group students were invited for semi-structured individual interviews. For interviews and group discussions, we printed out students’ survey results and brought them to the sessions. We asked students to review their responses and initiated questions based on their answers, such as “You chose to reject this plan. What was the most important perspective to make your decision?”, “Why did you not support the politician’s ideas?”, “What changes would make this proposal more acceptable to you?”, etc. During the discussion, students elaborated on their thoughts and ideas in detail. Each focus group discussion and individual interview took 40–50 min. The conversations and interviews with students were video recorded and transcribed for data analysis.

### Data analysis

We conducted a thematic analysis to understand how students reviewed and discussed perspectives in their decision making. To understand the emerging insights and themes in a specific case that has been researched, it is critical to recognize the interdependent relationship among different data sets and emerging themes through categorization (Williams & Moser, 2019). Based on the various data sets, we aimed to understand emerging themes of students’ thinking and decision making on the problem provided in the scenarios. At the beginning of the data analysis, four researchers in the research team individually reviewed the data to identify distinct themes. Following this, the team convened to share and compare their initial interpretations, refining, aligning, and categorizing the key ideas. Most of the key ideas from the individual analysis were overlapping and similar. There were different ideas, emphases, and interpretations when we brought forward our individual interpretations and key ideas. During our discussion on what differences were, where they emerged, and what data were related to the differences, we realized that the differences primarily lay in the terminologies used in our interpretations or the categories of key ideas. For instance, we discussed ‘empathy’ and ‘caring’ for students’ concerns about animals in the forests. After sharing the similarities and differences in the key ideas, we categorized them into themes. Thematization took several meetings to finalize as some key ideas could belong to a few themes and required us to go back to the data for cross-checking. While interpreting students’ survey data and focus group conversations, we examined the classroom data in order to understand students’ learning of scientific concepts and classroom discourse in comparison with the other data set. Based on reviewing various data sets, we developed themes that could explain the complexity of students’ evaluation of different perspectives and decision making on forests and land use. The themes arising from the data were (1) the priority of the environment, (2) mis/distrust toward politicians’ decisions in the community, and (3) empathy and altruistic thinking through perspective taking. In the following section, we share the results of students’ decisions on the scenarios and justifications that were extended with individual and group discussions for thematization.

## Findings

### Results of scenario surveys

The initial scenario survey (Appendix A) introduced diverse stakeholders’ views to students. Politicians suggested the Park proposal with economic growth, tourism, and revenue generation, which could benefit education and social welfare. Ecologists and biologists emphasized the value of biodiversity in the forests. Environmentalists were concerned about deforestation and climate change. A group of sociologists expressed their concerns about the displacement of communities and loss of community culture around the forests. Parents supported the park, expecting more opportunities for family leisure and the development of children’s athletic skills.

Students (*N* = 23) were asked to choose the level of importance of these ideas. Students’ responses to stakeholders’ ideas can be summarized in Table 1.

**Table 1:** Students’ responses in the initial scenario

	Very important		Important		Neutral		Not important		Not important at all	
	Frequency (*n*)	Percent (%)	Frequency (*n*)	Percent (%)	Frequency (*n*)	Percent (%)	Frequency (*n*)	Percent (%)	Frequency (*n*)	Percent (%)
**Politicians** on the development of economy, tourism, and revenue generation for education and social welfare			15	65.2	7	30.4			1	4.3
**Ecologists & biologists** on value of biodiversity	11	47.8	5	21.7	7	30.4				
**Environmentalists** on climate change and deforestation*	19	86.4	3	13.6						
**Sociologists on** displacement of communities, loss of culture, etc.	12	52.2	8	34.8	3	13.0				
**Parents** on family leisure and sports	3	13.0	11	47.8	8	34.8			1	4.3

*The total number of the frequencies is 22. One student did not respond to the question

Most students indicated that the stakeholders’ ideas were very important, important, or neutral for understanding issues and making decisions. Specifically, 86.4% of students rated environmentalists’ perspectives as very important, 52% rated sociologists’ perspectives as very important, 47.8% rated ecologists’ and biologists’ perspectives as very important, and only 13% rated parents’ perspectives as very important. Notably, no students rated politicians’ perspectives as very important. Overall, students prioritized environmentalists’ perspectives the most, while politicians’ and parents’ perspectives were considered the least important.

After reading the stakeholders’ views and choosing the levels of importance of their views for decision making, students were asked to either support or reject the proposal and write down reasons for their choice. For the first Google survey, 5 out of 23 students supported the plan, 12 students were opposed, and 6 students answered they were not sure about their decision. Among the 6 students (not sure), 4 students didn’t explain the reasons for their decision. 2 students explained that they couldn’t decide because there were both pros and cons of the plan and indicated the difficulties of decision making. Their reason to support the plan was that there were positive outcomes of the land use such as ‘they would get to learn something new or maximize the use of the empty land’, yet, some students put a condition of support, for example, as long as ‘the plan does not make climate change worse’. 7 out of 10 students who were opposed to the plan mentioned environmental aspects (ecosystem, climate change, plants and animals, Planet Earth, etc.). Among them, 4 students were especially concerned that it would damage the town, city, and province and people’s lives in them and 2 students mentioned that the park would be good only for certain people including politicians.

After the first survey, conversations with focus group students were conducted where students shared their ideas about the scenario and their decisions further. Students explained why the proposal was acceptable or not and what needed to be thought through before making a decision. Based on these conversations, we revised the plan (developing accessibility to the park through public transportation, how to protect natural habitats and peoples’ communities around the region, how to use the revenue for social welfare, etc.) (Appendix B) and provided the second survey (Table 2). Two students did not take the second survey, thus, we excluded their results in Table 2. 9 out of 21 of the students accepted the revised proposal, 1 student rejected it, and 11 students said they were not sure and could not decide. Some students changed their decisions (Table 3). 5 students changed their decision from reject to accept, 2 students changed from not sure to accept. 3 students changed from yes to not sure and 4 students from reject to not sure. Students who rejected the initial proposal changed their decision to accept or not sure, can decide.

**Table 2:** Students’ decisions on scenarios (*n* = 21*)

Students’ decision	The initial proposal	The revised proposal
Yes, Support	5	9
No, Reject	10	1
Not sure. Can’t decide	6	11

*We excluded the students who missed either one of the two surveys

**Table 3:** Changes in students’ decisions in the 2nd scenario (*n* = 21*)

Accept (Total = 9)		Reject (Total = 1)		Not sure (Total = 11)	
Accept to Accept	2	Accept to Reject	0	Accept to Not sure	3
Reject to Accept	5	Reject to Reject	1	Reject to Not sure	4
Not sure to Accept	2	Not sure to Reject	0	Not sure to Not sure	4

*We excluded the students who missed either one of the two surveys

Students who accepted the proposal explained that ‘the developers took responsibility for the land they were using,’ ‘public transportation would be better for the atmosphere’, and ‘people in Alfa would get benefits more than outsiders’. Students who were not sure about this plan explained that both advantages (economic benefits) and disadvantages (environmental issues) were clear and reasonable and that made their decision hard. They explained, “*I think of the good things and the bad things and I can’t decide because I can only choose one and not both”*,* “it could hurt the environment but it also has many benefits for the people of Alfa and other town for a number of reasons including tourism and jobs”*, etc. One student who rejected the plan in both scenarios emphasized the concern about cutting trees. He wrote, “*why cut trees? If anything they like to build*,* it should not be around the forests*”. During the interviews after the second survey, students said the revised proposal was better than the initial proposal. However, they emphasized that the approach needed to be careful around the environment and people near the forests.

### Results from conversations with students

Students had opportunities to explain their ideas on reviewing the stakeholders’ positions and reasons for their decision making in the Google surveys, yet, their explanation was brief. When we engaged students in the focus group conversations and individual interviews, they explained their perspectives, wonders, and questions further. Certain ideas were extensively and distinctively emphasized throughout the conversations. We thematized students’ ideas as follows.

### Environment first

#### Theme 1. The environment was the first priority for students’ decision making

In their written responses to the scenario surveys and discussion on the Park proposal, students emphasized the importance of environmental impacts in the decision-making process. Their awareness and concerns about the environment were shared as the priority of their decision-making during their conversations. Among diverse stakeholders’ views, students supported the environmentalists’ perspectives and concerns around deforestation and climate change the most, as they believed that building the Park would damage the environment and the Earth.

If the person was still wanting to build the amusement park, then I think they probably want to be worried about the environment then.…The air quality could be really bad, if they took down all the trees. (Mary)

If we don’t have an environment, we don’t have the world. Yeah, right. It’s like if we have the environment, then if the environment is bad, we can’t do these things anyways, so might as well preserve it first and find different ways. (Anna)

We only have one earth…If they cut down more trees, eventually they’ll leave no trees because they keep cutting down more trees than we can replant. (Jen)

Students were not opposed to the ways of making money and economic development, but they questioned how it would devastate the environment in the long run, thus, decided to reject the plan.

Have the money, live a better life. What better life can the people get if, like the earth is continually getting hotter and also colder and like more natural disasters? (Anna)

Survival or money! Would you rather have like $10 million and not live for the next day? (David)

During the focus group conversations, we asked students to rank the perspectives based on priority for their decision making. All students ranked environmental issues and climate change on the top and explained that ‘climate change is a serious problem’ when they explained their reasoning for ranking. Students who supported the Park proposal also suggested that people needed to find an alternative solution such as looking for areas of dead trees impacted by wildfires or mountain pine beetle attacks so that they didn’t need to cut healthy trees and destroy natural habitats.

Forest fires would make the land empty and abandoned, so it might be ok to build the park. (Chris)

The eagerness to compromise the plan with environmental awareness, care, and economic benefits was evident in their reasoning. They inquired for more information to make decisions.

I need information about the park … Like how it’s being run, how it would work there or where it would be. (Mary)

I need information to accept the proposal like, what animals live there. (Jen)

It was clear that students prioritized the environment first and believed decisions needed to be made when there was no damage to the environment and climate change.

### Mis/Trust toward stakeholders

#### Theme 2. Students were skeptical about politicians’ perspectives and engagement in decision making for the community

During the conversations on the multiple perspectives that we provided, students expressed their doubts and mistrust of politicians’ perspectives and trust toward environmentalists. Students did not trust what politicians suggested in the proposal.

Creating a strong economy is good and making it (the town) world famous would bring some tourists from other provinces like BC. But I am not sure about them donating stuff to good causes though, because a lot of the times politicians say something that they will do but in the long run they don’t actually do it. (Sam)

During the conversation, some students mentioned the benefits that politicians proposed such as developing education and social welfare with the company profit taxes, yet, other students didn’t trust that the politicians would keep their promises. Furthermore, students explained that the decision making from the government was to generate monetary gains, power, and votes from the public, not for people or the environment.

We have altered the structure of the Earth so much. Our Earth is dying and we are the cause of it and it doesn’t look like it’s going to stop right here. Because politicians, like governments … umm, with a political leader, they are often in it just for the money. In it for what they think is the greater good, it’s often not for the public. It’s often a very different vision for the public. (Sam)

If more people live there (because of the Park), then the government would raise taxes one day and inflation would happen. (Jen)

The constant comparison between the politicians and the environmentalists was driven by the fact that politicians supported the Park and the environmentalists did not. We asked what if the same suggestion was made by the environmentalists, not politicians and if they would agree with the suggestion. Then students responded that they would support the ideas as follows.

Researcher 1: Which information or knowledge is more important?

David: Environmentalists have better ideas about natural resources.

Researcher 2: why?

Anna: environmentalists, yes!

David: They just make better decisions about natural resources.

Chris: 100%, 100%. Politicians probably built a whole city over and, vote for me. I built the whole city over this and you can all get out for fun.

David: Yeah, that sounds really horrible.

Researcher 1: What if we scratch the politicians and put environmentalists here?

David: very important.

Researcher 1: Do you think it’s more believable?

Chris, David: Yeah (Anna: nodding).

Mary: Then it would sound more real coming from them [environmentalists]. Politicians are not gonna know as much as the environmentalists and all.

Chris: Some politicians lie to get what they want… Coming from a legally licensed environmental specialist, it would be like a lot more trustable cause they are like, they want to help the environment…. I would support this if it was more like (building) an environmentalist station, because that way the people who build it, they would be ordered by the environmental specialist.

Students in the dialogue above were skeptical about politicians’ perspectives and decisions. The mention of environmentalists being “legally licensed” indicated that the environmentalist perspectives entailed a sense of authority and knowledge about the environment. They showed willingness to alter their stance because environmentalists’ knowledge would translate to actions intended to help the environment in the long run.

### Altruistic thinking for others

#### Theme 3. Students demonstrated empathy and altruistic thinking after reviewing stakeholders’ perspectives

Not only was Alfa [the town where the Park was proposed] located in the province of Alberta, but its information was situated in reference to the city that students live in. For instance, the second survey stated that ‘Alfa was about a 100 km drive from your city’. This allowed the students, as residents of the city and the province of Alberta, to find relevance and connection to the issues presented. As students themselves were not the residents of Alfa, they believed the decision should be made for people residing in the town of Alfa, and not for themselves living in a closeby city. They expressed concerns about how it would affect people’s lives in the area of Alfa, especially Indigenous peoples near the forests. Students questioned as to why the proposal talked about the benefits of the Park for people who lived in their city, and not the people who lived in Alfa, where the Park would be built. For instance, the students indicated disapproval when the survey suggested that the Park would make cities close to Alfa a famous tourist spot as well. For the students, the proposal appeared to be all about “us” rather than “them”; *them* referring to the individuals residing in Alfa, who might not even want the park in their town in the first place.

From the sociologists’ perspectives in the scenario, the potential displacement of homes, including Indigenous people in the community, was mentioned. Students discussed safeguarding the rights, land and values of the Indigenous communities and emphasized it as a priority for their decision making.

There are lives, cultures, communities at stake here. (Sam)

The Indigenous people are so well with the environment. They don’t cut down many trees. They only cut down the trees, and they use everything. They are known for protecting the environment and not killing animals just for meat, and then get rid of the rest. They use everything. They [the company] would be like, we are good because we are honoring Indigenous people. …[They] are cutting down trees that’s ironic to Indigenous culture. (Chris)

It’s like whenever climate change affects different people, groups or different areas. More than others, like whenever, like hurricanes are really bad because of climate change, then like it affects homeless people the most or people who have a lot less money than the rich areas. So, I think that climate change will affect the indigenous people over there more. Rather than reverse! (Mary)

It was evident that students believed that they needed to respect others’ experiences, rights, and lives that might get affected by their decision making. Students brought forward the right decision with morality in their discussion.

## Conclusion & discussion

### Negotiating perspectives for “right” decisions

Students’ perspective taking was present during examining and evaluating the content and context of scenarios for their decision making. Scenarios provided students with diverse stakeholders’ views to critically reflect and share their own perspectives. Students brought their own knowledge, experiences, feelings, and values to examine stakeholders’ perspectives and situations in the scenarios. In the midst of exploring multiple perspectives, students explored the benefits and negative consequences of the proposal, which challenged their decision-making. In the surveys, some students were indecisive by indicating both the benefits and drawbacks of the plan. They also raised skeptical questions about politicians’ perspectives. A few students explicitly stated that politicians’ decision making was not for public goods but for the political purpose and power. Even though others did not state reasons for their skepticism, they expressed the same concerns about politicians’ views. For the revised proposal in the second survey, more than half of the students answered they couldn’t decide because of the complexity of the issue. They acknowledged the difficulties of decision making where they could identify both the positive and negative outcomes of the situation. Students also required more information that might help their decision making further. The scenarios with diverse perspectives provided students opportunities to develop socioscientific reasoning abilities with the complexity of perspectives, skeptical questions and requesting further inquiry for problem solving (Kinslow et al., 2019; Sadler et al., 2011).

Despite the complexity of perspectives in decision making on problems, it was clear that the environment was a first priority in their decision making, overruling other benefits such as economic development or utilization of the facilities. They were eager to find alternative solutions to protect the environment while proceeding with the project. For instance, they suggested building the park in a barren area of dead or burnt trees. Students also discussed the necessity of cutting trees for lumber resources and the importance of protecting forests to combat climate change and ensuring oxygen supply for life. They suggested ‘cut only a small number of trees, not many’, or ‘cut only small trees’ in order to reduce the impact of logging. Students’ science knowledge and understanding of deforestation and reforestation were intermingled with the complexity of perspectives in their reasoning and decision making. Negotiating different knowledge and perspectives, students prioritized the environment as the central focus of their decision making.

The complexity of diverse knowledge and value systems makes everyday decision making challenging and controversial in social contexts. The involvement of human subjectivity, such as personal and social perspectives, ethics and values, are inevitable for one’s reasoning and problem solving (Sadler, 2004; Zeidler & Sadler, 2008). Students in this study acknowledged the challenge of decision making with diverse perspectives and knowledge systems, while also being conscious of what consequences their decision making could bring to other human and non-human beings. Righteousness was often addressed in students’ decision making and justification. Students were empathetic towards animals whose habitats would be destroyed because of deforestation. Students explained that a decision should be made for people who live in the region, not visitors or outsiders. They said that asking people to move out of their communities was unjustifiable. During the conversations, students often discussed the idea that Indigenous peoples had lived in the forests for hundreds of years and that building the park would not help them. They doubted politicians, who often don’t keep their promises to others; thus, they disregarded politicians’ views. Students’ personal beliefs, emotions, skepticism, and value judgment were explicitly related to moral righteousness in their conversations.

In this study, we did not particularly emphasize morality and ethics in the scenario. In the first scenario, we introduced what is currently being discussed in the local community regarding forests and land use. However, environmental issues are often intertwined with social justice and morality by nature, making it indispensable to incorporate righteousness into their reasoning. Researchers explain that children’s moral motivation—willingness to prioritize moral concerns over attractive non-moral values—is intrinsic, and children are inclined to do the right thing (e.g., Nunner-Winkler, 2007). They also explain that children’s moral motivation is not determined by early childhood experiences but develops over the course of time with complexity of personal and sociocultural relationships, norms and values. This means that students’ moral motivation changes based on later experiences and other values such as jobs, economy, politics, and the like, and they re-evaluate their commitment to moral decisions (Nunner-Winkler, 2007). This raises pedagogical challenges in developing knowledge and skills of socioscientific and environmental decision making in classrooms. Students’ decision making in this study was heavily focused on concerns about the environment and morality with a minimal engagement of scientific knowledge. There was not a clear conflict between direct self-interests and moral motivation in the scenarios. That is, even though students rejected the plan based on moral values, that would not bring any direct negative consequences to their lives. Having the Park built or not would not make any difference in their own lives, so making decisions based on moral motivation was not as difficult as they were not required to give up their own needs and wants. With this limitation of the scenarios in the study, we cannot confirm that students’ morality overruled the complexity of perspectives and self-interests or any disciplinary knowledge of science. Yet, we acknowledge that students paid attention to the environment, other beings, and the importance of Indigenous peoples’ community and culture in their negotiation of perspectives and decision-making as ‘the right thing to do.’

### Pedagogical implications

The topic of the environment and ecosystems has been one of the big ideas in the science curriculum. With the growing concerns of climate change locally and globally, climate change has become an explicit learning objective in the new science curriculum in the province (Alberta Education, 2021). Yet, as discussed in the existing literature, climate change education in schools, focusing on science content, has not been effective in bringing forth the complexity of knowledge systems, decision making, and action holistically. Human subjectivity, such as students’ experiences, emotions, and value judgment, has not been thoughtfully addressed due to various constraints of science curriculum. However, studies show it is critical for students to understand the complexity of socioscientific and environmental decision making. They explain that science alone does not contribute to one’s environmental understanding, decision making and action (e.g., Sternäng & Lundholm, 2012); yet, the gap is still ongoing in the school curriculum. Encouraging students to examine diverse perspectives and the challenges of decision making on the issues is fundamental for socioscientific and environmental reasoning. Students in this study explicitly showed awareness of the local environment and connections to climate change problems. Students’ perspectives were intertwined with diverse experiences, knowledge, and values, yet, they presented what they needed to value for decision making by examining the consequences of certain decisions and actions on others. Students’ understanding, experiences, and perspectives need to be invited to classroom learning to develop students’ scientific literacy Vision III.

We acknowledge the effectiveness and challenges of a scenario-based approach to bring forth perspective taking in students’ decision making. Scenario-based approaches that reflect local contexts provided the students with opportunities to reflect on their own understanding of local situations. The scenario in this study encouraged students to examine their experiences, awareness, and concerns about climate change issues with the current challenges of wildfires in the province. It also developed their conversations around the Truth and Reconciliation Commission in the current society. Indigenous peoples’ approach to the land and decision- making was valued. We further questioned how scenarios could impact students’ reasoning and decision making in diverse ways. Arvai et al. (2004) explained that there are certain guiding principles in scenario making that help students’ decision making on complex environmental problems, that is, availability heuristic, representativeness heuristic, anchoring with insufficient adjustment, and affective judgment. They explained how problems are framed based on these heuristics and would guide students’ decisions in certain directions, sometimes with biases. For instance, students make decisions on the urgency and availability of the current problems (availability heuristic), for instance, wildfire issues are more critical and urgent than typhoons for students in Alberta or acid rains are not urgent or risky as they are not present in the current time. How the problem is presented (representativeness heuristic) and which information is provided for problem solving (anchoring with insufficient adjustment) are also crucial to guide decision making. It was clear that scenarios in this study developed students’ skepticism and inquiry for further information for decision making. Lastly, affect heuristic is when students rely on emotions, which often impact one’s evaluation of problems. In this study, students’ empathy toward animals losing their habitats and Indigenous peoples’ relocations was often discussed. These heuristics urge teachers to thoughtfully reflect on the structure and content of scenarios to develop students’ understanding, critical reasoning, and decision making skills.

Another pedagogical question was about the lack of scientific knowledge in students’ reasoning and decision making as discussed in many previous studies. In students’ discussion and decision making in this study, scientific knowledge from the curriculum was minimal. They mentioned the loss of natural habitats, animal populations, and oxygen from trees; yet, the connection between the science content of trees and forests and environmental issues was not explicit. With the scenarios we provided to students, students emphasized their experiences, perspectives, and values for problem solving. This was rather anticipated, yet, it led us to question in what way scientific knowledge would be connected to students’ reasoning in more impactful ways and what would be the learning outcomes of socioscientific and environmental reasoning in a holistic-interdisciplinary approach. As climate change has been addressed mostly in the science curriculum (Rousell & Cutter-Mackenzie-Knowles, 2020), science tends to be the main focus. The complexity of everyday decision making on climate change issues often minimizes the engagement of disciplinary knowledge of science. This brings science teachers some degree of dilemma and dissatisfaction in teaching complex decision making on climate change. This study was also conducted in a science classroom, and we examined the connection between science knowledge and socioscientific reasoning with perspectives. We included scientists’ (biologists and ecologists) perspectives on the forests, such as biodiversity and ecosystems, yet, we also experienced the dilemmatic situation of missing scientific knowledge in students’ discussions and questioned how to develop students’ knowledge building and decision making in integrated ways. We will continuously examine how students develop, explore and negotiate diverse knowledge systems and perspectives for decision making and how teachers can help students with the complexity of decision making going beyond disciplinary boundaries in future research.

### Limitations of the study

Environmental decision making is inherently complex and shaped by specific times, places, and community contexts. However, the scenarios provided lacked detailed information about these situated contexts. In hindsight, providing students with more comprehensive information about multiple perspectives could have significantly enhanced their ability to evaluate the complexities of the issues and navigate the decision making process more effectively. In this study, some students reported insufficient information, which may have contributed to their uncertainty as they struggled to fully understand the nuances of the perspectives presented in the scenarios.

Furthermore, students’ perspective taking and decision making are influenced by their understanding and experiences in the classroom, home, and community. However, the survey questions used in this study did not adequately examine what students had learned or discussed with their teachers and parents regarding local forests and land development. To address this gap, future research should consider redesigning scenarios and surveys to explore the connections among students’ prior knowledge, perspectives, and decision making processes more explicitly.

## Data Availability

The datasets used and analysed during the current study are available from the corresponding author on reasonable request.

## Appendix A

The survey was conducted using a Google Form, incorporating visual images to aid students in understanding the situations. Below is the content only.

### Amusement park survey

Welcome to the Amusement Park Survey! We invite you to take part in this survey, which aims to gather your valuable insights and opinions regarding a hypothetical business plan proposed for the town of Alfa.

In this survey, you will be presented with a fictional business plan that outlines a potential new venture in Alfa. This plan may cover various aspects, such as the type of business, its location, its impact on the local economy, and how it aligns with the community’s values and needs.

Your feedback is essential in helping us assess the potential benefits and concerns associated with this hypothetical business plan. We want to ensure that any future business ventures in Alfa contribute positively to the town’s growth and prosperity while considering the interests and well-being of its residents.

Please take a few moments to share your thoughts and opinions on this hypothetical business plan. Your contribution is invaluable in helping us make informed decisions about the future economic landscape of Alfa.

Thank you for your participation!

******.

Please read the following scenario carefully and answer the questions. Alfa is a small town located between Edmonton and Jasper. There are many lakes, forests, and animal habitats in Alfa. This town is well known for its rich biodiversity, and many endangered species live in this region. Thus, scientists value this place and come to conduct scientific research on its ecosystem.This town also has a lot of natural resources (e.g., trees, water, coal, natural gas, and minerals) on the land and underground. Thus, people say there is great potential for the economy in Alberta.Many of the communities also reside around the area. There are many cultural groups including Indigenous communities living there for hundreds of years.Recently, as part of the regional development plans for the next 10 years, a company has submitted a plan to build a huge resort town for multi-use facilities (skiing, snowboarding, tubing, etc. in winter; hiking, biking, zip lining in summer) in Alberta, called AlbertaLand Park. Here is the summary of their suggested plan. Deforestation (cutting many trees) is necessary to secure enough space for rides, restaurants, and other facilities in AlbertaLand Park.

The company will collaborate with local mining companies to extract the natural gas or coal to supply the energy (gas and electricity) for AlbertaLand Park. They can also sell the energy to develop facilities and infrastructures.

The company requires some start-up funding from the Alberta Government to build roads and irrigate water from the local rivers and lakes. They will pay back to the government after selling the energy to make profits.

They will create a lot of jobs for people living around Alfa.

AlbertaLand Park will attract many tourists and make the town world famous in the next five years.

Based on the business plan summarized above, there are various opinions from various stakeholders. In the next section, you will read their ideas one by one and rank them according to their importance for consideration.

POLITICIANS: Politicians believe that this business plan will turn Alfa into a world-renowned town.

The company will pay a lot of taxes and make donations to develop education, social welfare, health care systems, etc. AlbertaLand Park will create a stronger economy in the province.

Please indicate how important these arguments are: Not important, important, neutral, important, very important. Explain why you chose the stance in the above question.

ECOLOGISTS AND BIOLOGISTS: Ecologists and biologists suggest reservation of the land in Alfa because it has a lot of value in biodiversity.

Please indicate how important these arguments are: Not important, important, neutral, important, very important. Explain why you chose the stance in the above question.

ENVIRONMENTALISTS: Environmentalists argue that climate change is becoming a serious problem, and deforestation has made climate change worse for many years. There has already been a lot of loss in forests from wildfires, logging, mining, etc., around the Rockies.

Please indicate how important these arguments are: Not important, important, neutral, important, very important. Explain why you chose the stance in the above question.

SOCIOLOGISTS: Sociologists suggest that displacement of local people in that region will be problematic. These people may lose their homes, communities, and cultures.

Please indicate how important these arguments are: Not important, important, neutral, important, very important. Explain why you chose the stance in the above question.

PARENTS: Parents living in Alfa support the idea of having the resort and sport facilities in their town as it gives them a place to entertain their children. They believe this will provide children with many opportunities to explore winter and summer sports.

Please indicate how important these arguments are: Not important, important, neutral, important, very important. Explain why you chose the stance in the above question.

Your Opinion.

In this final question, we ask your opinion about the proposed business plan for Alfa.

Would you support the plan? Or would you reject the plan? Please explain your reasons.

## Appendix B

The survey was conducted using a Google Form, incorporating visual images to aid students in understanding the situations. Below is the content only.

### About the town of Alfa

There are many lakes, forests, and animal habitats in Alfa. This town is well known for its rich biodiversity, and many endangered species live in this region. This town also has a lot of natural resources (trees, water, coal, natural gas, minerals) on the land and underground, thus people say there is great potential for the economy in Alberta. Many of the communities also live around the area. There are many cultural groups including Indigenous communities living there for many years. Recently, there was a plan submitted to build AlbertaLand Park, a resort with multi-use facilities.

Please read the following scenario carefully and answer the questions.

You all expressed several concerns, leading to the rejection of the previous plan. This time, a new company has brought a proposal. Here is their suggested proposal:

1. Pine beetles attacked part of the forests in Alfa. Due to this attack, several trees have died within a short period of time. Pine beetles eat the trees from the inside (as shown in the photo below). Their attacks can spread to several other trees if the attacked trees are not cut down or taken care of. The company will cut the infested rees first.
2. The company plans on inviting Indigenous peoples for advice on development of the resort town. They plan on building an information center to educate visitors about the history and different cultures of Alberta (as shown in the photo below).
3. The company will take the responsibility to build a natural gas site (as shown in the photo below). This will supply energy not only to the resort but also to the people in the town. The company will also pay taxes to the town for using the land and their resources. The tax money collected from the company will be used to build schools, hospitals, roads, and the public transportation in Alfa.
4. Once AlbertaLand Park is doing well, and after the company has made some money, they will use that money to develop high speed trains to AlbertaLand. These trains will increase tourism to Alfa and encourage people to use the train instead of driving their own cars to visit AlbertaLand.
5. The company will hire workers from Alfa and from Edmonton. They want to help these people find jobs so they can earn money for themselves and their families.

- Do you think this proposal is acceptable and why? Explain your thoughts.
- Do you think this proposal is not acceptable and why? Explain your thoughts.
- If you are not sure, you can’t decide, why? Explain your thoughts.
